# Reduced Levels of miR–28 and miR–200a Act as Predictor Biomarkers of Aggressive Clinicopathological Characteristics in Gastric Cancer Patients

**DOI:** 10.31661/gmj.v8i0.1329

**Published:** 2019-01-25

**Authors:** Farhad Jeddi, Shahriar Alipour, Nowruz Najafzadeh, Mehdi Dadashpour, Farhad Pouremamali, Mohammad Reza Sadeghi, Nasser Samadi, Narges Soozangar, Amir Mahdi Khamaneh

**Affiliations:** ^1^Research Laboratory for Embryology and Stem Cells, Department of Anatomical Sciences and Pathology, School of Medicine, Ardabil University of Medical Sciences, Ardabil, Iran; ^2^Department of Biochemistry, Faculty of Medicine, Urmia University of Medical Sciences, Urmia, Iran; ^3^Department of Medical Biotechnology, Faculty of Advanced Medical Sciences, Tabriz University of Medical Sciences, Tabriz, Iran; ^4^Department of Molecular Medicine, Faculty of Advanced Medical Sciences, Tabriz University of Medical Sciences, Tabriz, Iran; ^5^Department of Biochemistry, Faculty of Medicine, Tabriz University of Medical Sciences, Tabriz, Iran

**Keywords:** Biomarkers, Gastric cancer, miRNA–28, miRNA–200a, Quantitative Real-time PCR

## Abstract

**Background:**

MicroRNAs (miRNAs) play critical roles in different pathological processes including cancer development and progression. To find novel molecular diagnostic and prognostic markers and promising therapeutic tools for gastric cancer (GC), we aimed to investigate the relationship of the expression levels of miR–28–5p or miR–200a–3p with the clinicopathological criteria and to explore their impacts on the progression of human GC.

**Materials and Methods:**

Quantitative RT–PCR was performed to analyze miR–28 and miR–200a expression in 60 GC and 60 non–GC tissue samples.

**Result:**

Our results revealed that the expressions of miR–200a and miR–28 were significantly downregulated in GC in comparison with non– GC tissues. Tumors with low miR–28 expression had larger tumor size, more advanced histological grade, and a higher incidence of lymph node and distal metastasis than the tumors with high miR–28 expressions. Furthermore, receiver operating characteristic (ROC) analyses demonstrate that the expression of miR–28 is a predictive biomarker allows predicting the histological grade, tumor size, and occurrence of nodal and distal metastases. We also found a significant inverse association between miR–200a expression and the rate of lymph node metastasis (p = 0.010, r = –0.334).

**Conclusion:**

Our findings suggest that the miR–28 and miR–200a have tumor–suppressor functions and may be considered as potential biomarkers for gastric cancer diagnosis and prognosis.

## Introduction

As the fourth most common cause of malignancies, gastric cancer (GC) represents the second reason of cancer death globally [1]. Most GC patients are identified at advanced stages of the disease, with local invasion or tumor metastasis and poor overall survival rate. However, if GC is detected in early stage, the survival rate of patients increases to over 90% [2, 3]. The prognosis of GC is influenced by several biological variables and the combination of molecular alterations may contribute to the aggressive progression of GC [4, 5]. However, the precise molecular mechanisms involved in the progression and carcinogenesis of GC have not yet been completely characterized. Therefore, it is extremely necessary to identify novel molecular diagnostic and prognostic markers to improve the clinical prognosis of GC patients as well as to enhance the efficiency of treatment strategies.MicroRNAs (miRNAs) are a category of endogenous, small (18–24 nucleotides), noncoding RNAs that primarily function as post-transcriptional regulators of protein-coding genes through binding to the untranslated regions (UTRs) at the 3’end of downstream mRNAs [6]. Recent studies have strongly supported the notion that microRNAs have critical roles in fundamental biological events, comprising cell proliferation, differentiation, and apoptosis, as well as different pathological processes such as carcinogenesis, tumor angiogenesis, and invasion [7-9]. More than fifty percent of microRNA–coding genes are anchored in the fragile regions as well as in genomic are as affected in various cancers [10], proposing the impacts of miRNAs in the pathogenesis of human cancer. Several tumor–associated miRNAs have been shown to be dysregulated in GC. For example, in a systematic review of 14 miRNA expression profiling studies, Shrestha *et al*. reported 352 miRNAs which dissimilarly expressed in participants with and without GC [11]. They introduced some of the candidate miRNAs as efficient biomarkers and therapeutic targets in human GC.The potential role of miR–28–5p and its mechanism have been studied in colorectal [12], ovarian [13], and VHL–associated cancer [14]. Almeida *et al*. showed that mir–28–5p had a remarkably lower expression in colorectal tumor tissues compared with normal colon tissues. In addition, upregulation of mir–28–5p could increase apoptosis and reduce cell proliferation and migration in the colorectal cancer cells [12]. A growing body of evidence has shown that the miR-200 family, consisting five members (miR–200a, miR–200b, miR–200c, miR–141, and miR–429), may serve as tumor-suppressor miRNAs and replacement of these miRNAs has been indicated as a new therapeutic strategy in multiple types of cancers [15-17]. miR–200a functions as a suppressor of tumor cell growth, epithelial–mesenchymal transition (EMT) and cancer invasion/metastasis [18-20]. These observations led us to evaluate the expressions of miR–28–5p and miR–200a–3p in gastric adenocarcinoma, and to determine their clinicopathological significance. Our findings support the interpretation that upregulation of tumor suppressor miR–28 and miR–200a can be an intriguing possibility for future GC treatment.

## Materials and Methods

### 1. Patients and Sample Collection

This case/control study was performed on the patients with gastric adenocarcinoma (n=60) referring to Madani and Imam Reza Hospitals, Tabriz, Iran. Fresh endoscopic biopsies taken from 60 non-GC patients were also prepared as control samples. The tissue specimens were immediately frozen in liquid nitrogen as soon as possible, and subsequently stored at –80°C till further analysis. One piece of each sample was fixed with 4% paraformaldehyde and used for histological analysis.

### 2. Quantitative Reverse Transcription PCR Analysis (qRT–PCR)

#### 2.1. qRT–PCR Analysis of miR–28 and miR–200a Expressions

The relative expression levels of miR–28–5p and miR–200a–3p in both GC and non–GC tissue samples were analyzed by qRT–PCR method. TriPure Isolation Reagent (Roche, Germany) was applied to extract total RNA by physical disruption and Phenol/Chloroform methods. RNA from each tissue sample was reverse–transcribed to complementary DNA (cDNA) by using the miRCURY LNATM Universal cDNA synthesis kit (Cat No. #203301) on Thermocycler (Eppendorf). Briefly, 100 ng RNA was mixed with 2 μl of 5× reaction buffer, 1 μl reverse transcriptase, and up to 4.5 μl of nuclease-free water, incubated in PCR machine for 60 min at 42˚C, 5 min at 95˚C and then, immediately cooled to 4°C. The resultant cDNA was diluted 20 times, and served as a template for miRNA qRT–PCR using miRCURY LNATMUniversal RT miRNA PCR SYBR® Green kit (Exiqon, Denmark) and primer sets (Product No. 204322 and 204707, Exiqon, Vedbaek, Denmark). Four microliters of diluted cDNA were used in each real-time PCR reaction, which contained 1μL PCR primer set and 5 μL PCR Master Mix to make a final volume of 10 μL. Amplification carried out under the following conditions: initial denaturation at 95 °C for 10 min, 40 cycles at 95°C for 10 seconds and then 60°C for 1 minute. The primer sequences were as follows: hsa-miR-28-5p (5′-AAGGAGCUCACAGUCUAUUGAG-3′); hsa-miR-200a-3p (5′-CATCTTACCGGACAGTGCTGGA-3′); hsa-miR-103 (5′-AGCAGCATTGTACAGGGCTATGA-3′).

#### 2.2. Normalization Method

The comparative CT method was performed to measure the relative expression levels of miR–28 and miR–200a in the GC and non–GC biopsy samples. All qRT–PCR data were normalized after subtracting the CT values of these miRNAs from that of miR–103 (Cat No. #204030) as an internal control (2^–ΔCT^ method, ΔCT = CT_miR–28 or miR–200a_ –Ct_miR–103_). Each measurement was performed in triplicate.

#### 2.3. Ethical Statements

This project was reviewed and approved at the Research Ethics Committee of Tabriz University of Medical Sciences (Reference number: 1394.618). All participants were asked to sign a written consent describing the study aims and the subsequent procedures.

#### 2.4. Statistical Analysis

The Mann–Whitney analysis was employed to evaluate the differential expression of miR–28 and miR–200a between GC and non–GC specimens. The statistical significances among clinicopathological characteristics and both miR–28 and miR–200a expression levels were assessed by Chi-square (χ2) test. All statistical tests were carried out by GraphPad Prism version 6 (GraphPad Software Inc., San Diego, California) or SPSS software version 11.0 (SPSS Inc., Chicago, Illinois). P value of less than 0.05 was considered as statistically significant.

## Results

### 1. Demographic Information

There were 40 males and 20 females in the gastric cancer group with the average age of 68.3 ± 12.9 years, ranging from 31 to 93 years. In non-GC patients, the mean age was 61.8 ± 15.1 years, the range of 23–84 years. The clinicopathological data of the participants are represented in Table 1.

### 2. miR–28 and miR–200a were Downregulated in Gastric Cancer Patients

**Figure 1:**
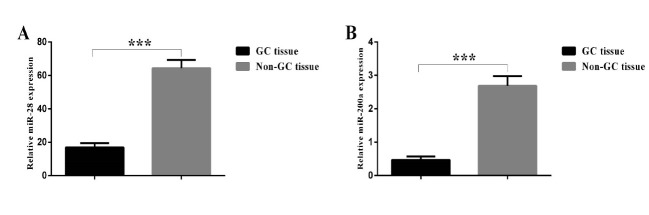
miR–28and miR–200a expression in GC and non –GC tissue samples detected by quantitative reverse transcription–polymerase chain reaction (qRT–PCR) analysis.

To explore the roles of the miR–28–5p and miR–200a–3p in the GC, the expression levels of these miRNAs were assessed by quantitative RT–PCR in all tissue samples. Our results showed that the expression levels of miR–28 (GC vs. non–GC: 16.877 ± 2.603 vs 64.329 ± 4.885, P< 0.001) and miR–200a (GC vs non–GC: 0.468 ± 0.105 vs 2.683 ± 0.293, P< 0.001) in human GC tissues were significantly lower than those in non–GC tissues (Figure 1). To determine the relationship between clinicopathological criteria and miRNAs expression, the median values of miR–28 (7.790) and miR–200a (0.227) expression were used as cutoff points for classifying all 60 patients with gastric adenocarcinoma into miR–28–low/high and miR–200a–low/high groups, respectively.

### 3. Downregulation of miR–28 Associates with Aggressive Progression of Gastric Cancer

**Figure 2:**
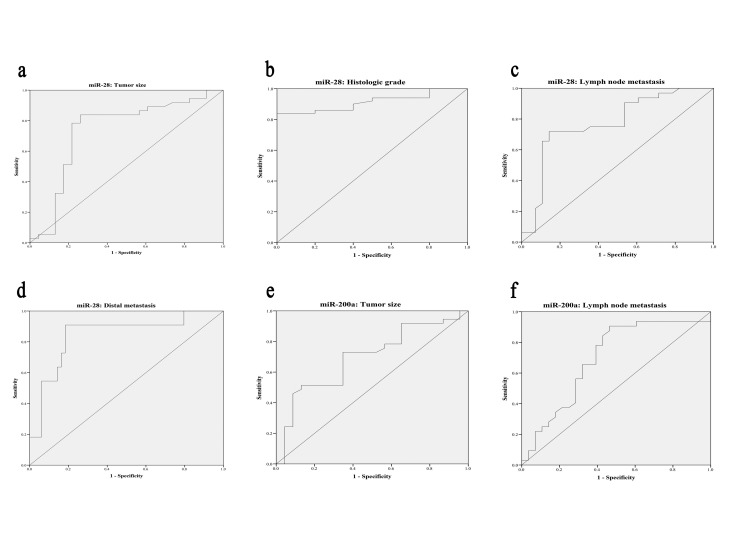
Diagnostic values of *miR–28* and *miR–200a* in GC. Expression of miR–28 was applicable molecular biomarker for prediction of tumor size (a), histological grade (b) and occurrence of lymph node (c) and distal metastasis (d). In addition, ROC curve analysis showed the potential of *miR–200a* level to predict tumor size (e) and occurrence of lymph node metastasis (f) in gastric adenocarcinoma.

The association between clinicopathological features and miR–28 expression is summarized in Table 2. GC patients with low expression levels of miR–28 had greater tumor size (p = 0.001), higher histologic grade (p = 0.004), and more frequently positive lymph node (p<0.001) and distal metastasis (p= 0.003). However, there were no significant correlations between miR–28 expression and other clinicopathologic variables such as sex, age, and smoking status (all p>0.05). Further statistical analysis showed considerable differences in miR–28 level between poor (high grade) and moderate (intermediate grade), as well as between well (low grade) and poorly differentiated tumors (all p > 0.05). Nevertheless, no significant difference was found in miR–28 expression between well and moderately differentiated adenocarcinomas (p> 0.05).Receiver operating characteristic (ROC) curve was also applied to survey the predictive value of miR–28 level in the GC patients for clinicopathologic criteria. ROC results suggested that miR–28 had the capability to predict tumor size, histologic grade and occurrence of lymph node and distal metastases (Table 3). The expression of miR–28 and tumor size yielded a significant AUC of 0.735 (95 % confidence interval 0.590–0.880; p=0.002) with a sensitivity of 83.8 %, specificity of 73.9 %, and optimal cutoff point of 4.11 (Figure 2a). AUC for the histological grade was 0.913 (95 % confidence interval 0.841–0.985; p<0.001) with an optimal cutoff point of 3.04, where the corresponding specificity and sensitivity were 100 % and 84.0 %, respectively (Figure 2b). The optimal cutoff values of miR–28 were 6.32 and 3.09 for predicting lymph node and distal metastasis with AUC values of 0.773 (95 % confidence interval 0.649–0.897; p<0.001) and 0.844 (95 % confidence interval 0.705–0.983; p<0.001), respectively. The corresponding sensitivity and specificity were 71.9 and 85.7%, and 90.9 and 81.6%, respectively (Figure 2c, d).

### 4. Association Between Clinicopathological Criteria and miR–200a Level in gastric Adenocarcinoma

To assess whether the downregulation of miR-200a expression in the GC tissues was associated with clinicopathologic features, the Chi-square (χ2) test was applied. The statistical analysis showed that the miR–200a expression only had an association with lymph node metastasis (p = 0.010). As shown in Table 2, the low levels of miR–200a expression were 65.6% (21 of 31) and 32.1% (9 of 28) in the patients with and without lymph node metastasis, respectively. Indeed, in GC tissue samples, the miR–200a expression was downregulated when lymph node metastasis was present. However, there were no remarkable associations among miR–200a levels and sex (p = 0.103), age (p = 0.587), tumor size (p = 0.065), histologic grade (p = 0.103), distal metastases (p = 0.741), or smoking status (p = 0.351). The prognostic significance of miR-200a was elucidated with ROC curves. Our results showed that miR–200a had the ability only to predict tumor size and lymph node metastasis (Table 3). The AUC values were 0.696 (95 % confidence interval 0.559–0.833; p=0.011) and 0.695 (95 % confidence interval 0.556–0.835; p=0.010) with cutoff points of 0.334 and 0.444, where the corresponding sensitivity and specificity were 51.4 % and 87.0 %, and 90.6 % and 53.6 %, respectively (Figure 2e, f).

## Discussion

Previous studies strongly support the notion that miRNAs, as post–transcriptional regulatory molecules, can target up to one–third of human coding genes and act as oncogenes or tumor suppressor genes [21, 22]. Thus, identification of novel and differentially expressed miRNAs should be considered as a topic of intense research in the cancer diagnosis, prognosis, and therapy. In our research, we aimed to investigate the expression level of miR–28 and miR–200a in the patients with gastric cancer and to determine their relationship with clinicopathologic parameters. Our results showed that miR–28 and miR–200a expressions in the GC tissue samples were considerably lower compared to noncancerous samples. Furthermore, the reduced level of miR–28 was significantly relevant to larger tumor sizes, more advanced histologic grades, and a higher incidence of lymph node and distal metastases in gastric adenocarcinomas. In addition, ROC results revealed that the expression of miR–28 was applicable molecular biomarker for prediction of tumor sizes, histologic grades, and occurrence of lymph node and distal metastases.Based on the tumor tissue and the cell type, miRNAs have oncogenic or tumor-suppressor functions [23-27]. miR–28–5p displays differential expression patterns and plays diverse roles in the development of human cancers [12, 13, 28-30]. For example, Xu *et al*. indicated that miR–28–5p was considerably overexpressed in ovarian cancer samples compared with adjacent non–malignant controls and its overexpression could enhance the ovarian cancer cell progression, invasion, migration, and proliferation [13]. Similarly, in glioma cells, Malzkorn *et al*. showed that miR–28 level was increased during glioma progression in the majority of investigated patients [28]. However, miR–28 was significantly downregulated in breast cancer cells, where it targets the 3′UTR of Nrf2 mRNA. In another study, downregulation of miR–28 was identified to be oppositely correlated with tumor metastasis, recurrence, and poor survival of hepatocellular carcinoma, indicating a tumor–suppressor function for this microRNAs [31]. Moreover, miR–28–5p was found to be downregulated in renal cell carcinomas [30] and colorectal tumors [12], while it’s in vitro upregulation could reduce invasion, migration, and proliferation of colorectal carcinoma. Accumulating evidence suggests that there is heterogeneity in miR–28–5p expression in various cancers, and investigation of miR–28 expression can be considered as a molecular signature to identify clinical and pathological prognostic factors. Our results also revealed a significant negative correlation between the incidence of lymph node metastasis and expression level of miR–200a. Moreover, ROC curve analysis showed the potential of miR–200a level to predict tumor size and occurrence of lymph node metastasis in the gastric adenocarcinoma. Other studies also showed low levels of miR–200a in tumor tissues and cell lines [16, 18, 19, 32]. Chang *et al*. found that members of the miR-200 family had significantly lower expressions in the GC tissues when compared with matched nonmalignant tissues [16]. In another study, Sun *et al*. also found similar results that miR–200a expression level was negatively correlated with tumor metastases in the ovarian tumors [32]. They found no remarkable relationship among the miR–200a level and tumor size, histological type, and grade. Gain and loss of function study by Pichler *et al* showed that downregulation of miR–200a-3p by using a specific miR–200a inhibitor led to increased expression of EMT–related genes in colorectal cancer cell lines [19]. They also demonstrated that lower levels of miR–200a were associated with poor survival in colorectal cancer patients. However, in a different study, the high level of serum miR–200a-3p was seen in epithelial ovarian tumors, where it correlated with histological subtype and stage [33]. The limitation of our study was that we could not analyze the association between overall survival and the microRNAs expression because it requires patients to be followed up to five years.

## Conclusion

Our results highlight the significance of the miR–28–5p and miR–200a–3p as potential biomarkers in the diagnosis and prognosis of gastric adenocarcinoma. These miRNAs may play tumor–suppressor functions in the carcinogenesis and tumor progression of gastric cancer through the regulation of cell proliferation, differentiation, tumor angiogenesis, and invasion. However, future investigations are necessary to identify the molecular mechanisms of miR–28 and miR–200a in the development and progression of gastric adenocarcinoma.

## Acknowledgment

This study was supported by the research fund of Ardabil University of Medical Sciences, Ardabil, Iran and Student Research Committee, Tabriz University of Medical Sciences, Tabriz, Iran (grant number: 95/81).

## Conflict of Interest

The authors declare no conflicts of interest.

**Table 1:** Summary of Clinicopathological Features of Patients withGgastric Cancer

**Clinical Pathological** **features**	**No. of Patients (%)**
**Sex**	
Male	40 (66.7)
Female	20 (33.3)
**Age (year)**	
< 65	20 (33.3)
≥65	40 (66.7)
**Tumor size (cm)**	
< 5	37 (61.7)
≥5	23 (38.3)
**Histologic grade of differentiation**	
Well	14 (23.3)
Moderate	36 (60)
Poor	10 (16.7)
**Lymph node metastasis**	
Present	32 (53.3)
Absent	28 (46.7)
**Distal metastasis**	
Present	11 (18.3)
Absent	49 (81.7)

**Table 2:** Association of miR-28 and miR–200a Expressions with Various Clinicopathologicalcriteria of Patients With Gastric Cancer

**Clinical Pathological** **Criteria**	**miR–28 Expression**		**P value**	**miR–200a Expression**		**P value**
	**Low (%)**	**High (%)**		**Low (%)**	**High (%)**	
**Sex**			0.587			0.103
Male	21 (52.5)	19 (47.5)		23 (57.5)	17 (42.5)	
Female	9 (35.0)	11 (65.0)		7 (35.0)	13 (65.0)	
**Age (years)**			0.277			0.587
< 65	8 (40.0)	12 (60.0)		11 (55.0)	9 (45.0)	
≥65	22 (55.0)	18 (45.0)		19 (47.5)	21 (52.5)	
**Tumor size (cm)**			0.001			0.065
< 5	12 (32.4)	25 (67.6)		15 (40.5)	22 (59.5)	
≥5	18 (78.3)	5 (21.7)		15 (65.2)	8 (34.8)	
**Histologic grade of differentiation**			0.001			0.492
Well/ Moderately	20 (40.0)	30 (60.0)		24 (48.0)	26 (52.0)	
Poor	10 (100.0)	0 (0.0)		6 (60.0)	4 (40.0)	
**Lymph node metastasis**			<0.001			0.010
Present	23 (71.9)	9 (28.1)		21 (65.6)	11 (34.4)	
Absent	7 (25.0)	21 (75.0)		9 (32.1)	19 (67.9)	
**Distal metastasis**			0.003			0.741
Present	10 (90.9)	1 (9.1)		5 (45.5)	6 (54.5)	
Absent	20 (40.8)	29 (59.2)		25 (51.0)	24 (49.0)	
**Smoking status**			0.756			0.351
Smoker	7 (53.8)	6 (46.2)		8 (61.5)	5 (38.5)	
Never–smoker	23 (48.9)	24 (51.1)		22 (46.8)	25 (53.2)	

**Table 3:** AUCs for ROC Curve Corresponding to the Diagnostic values of miR–28 and miR–200a in Gastric Cancer.

**Parameter**	**miR–28**				**miR–200a**			
	**AUC**	**Standard Error**	**95 % C.I.**	**P value**	**AUC**	**Standard Error**	**95 % C.I.**	**P value**
**Tumor size**	0.735	0.074	0.590–0.880	0.002	0.696	0.070	0.559–0.833	0.011
**Histologic grade**	0.913	0.037	0.841–0.985	<0.001	0.659	0.080	0.502–0.816	0.115
**Lymph node metastasis**	0.773	0.063	0.649–0.897	<0.001	0.695	0.071	0.556–0.835	0.010
**Distal metastasis**	0.844	0.071	0.705–0.983	<0.001	0.509	0.099	0.315–0.703	0.924

GC: Gastric cancer, ***: p< 0.001.

GC: gastric cancer, ROC: receiver operating characteristics.
